# Address to Michigan Dental Association

**Published:** 1866-03

**Authors:** J. A. Watling

**Affiliations:** The Retiring President


### ADDRESS TO MICHIGAN DENTAL ASSOCIATION.

BY J. A. WATLING, D.D.S.
The Retiring President.

Mr. President and Gentlemen: — Another year has rolled along upon the ceaseless wheels of time since we last met together; another year, fraught with our pleasures and sorrows, our toils, our thoughts, and our deeds, is gone. And as your retiring President, I cannot avoid the question, What have we done, as an association, for ourselves, for each other, and our *noble* profession, in the year that, for us as a nation, has so signally gone? Gone, with the tears of the bereaved, the groans of its dying, and graves of the dead, for the cruel war, which convulsed our country from its Atlantic to its Pacific shore, is ended, and other trembling nations *feel* that America *will* be *free*, her people the most enlightened, their principles the most exalted, their professions the most *advanced* and *perfected.* What have *we* done to elevate the standard of our chosen profession? to wrest it from the net-work of ignorance and empiricism that entangles it? I need hardly say that it gives me more than ordinary pleasure to note the increased fervor manifested in our association, and the general interest awakened all over the country, during the past year, resulting in the organization of *new* colleges and associations, which we trust shall serve as strong pillars whereon to build the structure of our brighter future. Another very encouraging fact is the increased number of students attending our dental colleges, and especially those from our own State, which far exceed all that have gone in time before. With reference to our failure to establish a dental department in the university of Michigan, I must attribute it in part to the want of that *zeal* and earnestness that must ever accompany all great and important undertakings. We must remember that no great end is accomplished without *great* effort; and, gentlemen, let me assure you that *this* is a *great* and *important* matter, both to us as a profession and to the medical department of the university; it being an experiment to determine whether *specialties* can be taught in harmony with general medicine. If it should prove successful, (which I believe it will, if ever tried), I hope the time will come when every regular medical school shall have its chair of dentistry, as I believe it to be the true way of teaching our science. Many of our best medical colleges are alive to the necessity of establishing chairs of specialties, and the subject of dentistry has already been agitated as one of the *most important.*

Then let us *persevere* and we may yet see the degree of D.D.S. conferred in our noble university before it is by other similar institutions; for do we not all believe that the dentist should have as thorough a medical knowledge as the oculist or aurist? And are we not ready and anxious to do with our might whatever we *can* do, that he may attain it? Those of you who were present at the meeting of the American Dental Association, in Chicago, must have been highly gratified with the interest manifested in our department by the general practitioner, and the more perfect recognition of dentistry as a very essential specialty of medicine. And now, gentlemen, whether or not the character of our profession and the interest thus manifested in it, be maintained, whether the benefits to be derived from it shall be perpetuated, depends upon *us*, both personally and as an association. Although we may still be groping our way in comparative darkness, yet rest assured that the day begins to dawn upon a higher and nobler professional standard. The sun of science, so long hidden under the vail of empiricism and ignorance, begins to break through, casting its vivifying rays upon us, and will continue to brighten and lighten our path, till we walk as in perfect day. The lovers of darkness, who were banded together to practice their arts of imposition and quackery, intent only upon filling their coffers at the expense and comfort of their patrons, already tremble at the steadily advancing step of progress. They are not here to-day; they have no desire to meet and take counsel with those who seek the happiness and welfare of humanity, the best interests and brightest prosperity of dentistry. We cannot go back for them. Do they care for improvement, and wish it success? Let them grasp the right hand of fellowship we now offer them; with us *toil* zealously and long; with us to reap in the glorious harvest-time. Let them unite with us in these associations, not for pecuniary benefit, but for mutual improvement, counsel and advice, that we may be the better able to serve those requiring our assistance. Let each member come forward and give freely any knowledge that may benefit another, however trifling he may deem it. And now, gentlemen, let me here impress upon your minds the urgent need we have, individually, of this unselfish interchange of thoughts and feelings. It is absolutely necessary that we each do what brain-work we can, and bring forward the result, though it be but a *mite*. I well remember a single idea I received at Saratoga, which paid me for my journey. Our thoughts are but atoms of the great world of thought; and whether they glimmer with a flickering light, or shine more brilliantly with the *true fire* of genius, they each have a part to perform, a mission to fulfill, in the elevation of our noble profession. Therefore let us each bring our thought—it may be just the *atom* most needed to make perfect the whole.

And now let me call your attention to one thing that I think shows degeneracy, rather than improvement, and most earnestly ask your assistance in a reformation. It is to the modern method of procuring business by *quack advertisements*, a method so derogatory to a true professional standard. Let us one and all *denounce* it, wherever found, whether emanating from young or old; in newspapers or in flaming handbills with *self-assumed* degrees. Let us teach the people that true merit needs no *self-puffing*.

Finally, let us steadily and unitedly work on; ever just and true to our patrons, and we shall be to our profession and *ourselves*. *Jan. 30th*, 1866.

